# Patterns and predictors of chronic opioid use in older adults: A retrospective cohort study

**DOI:** 10.1371/journal.pone.0210341

**Published:** 2019-01-11

**Authors:** GYeon Oh, Erin L. Abner, David W. Fardo, Patricia R. Freeman, Daniela C. Moga

**Affiliations:** 1 Department of Epidemiology, University of Kentucky, Lexington, Kentucky, United States of America; 2 Sanders-Brown Center on Aging, University of Kentucky, Lexington, Kentucky, United States of America; 3 Department of Biostatistics, University of Kentucky, Lexington, Kentucky, United States of America; 4 Department of Pharmacy Practice and Science, University of Kentucky, Lexington, Kentucky, United States of America; University of Sydney, AUSTRALIA

## Abstract

**Background:**

Given the controversy around the effectiveness of opioid treatment for chronic pain and the lack of detailed guidance for prescribing opioids in older adults, the objectives of this study were to estimate the trajectories and predictors of opioid use in older adults.

**Methods:**

Data were extracted from the National Alzheimer’s Coordinating Center (2005–2017). Group-based trajectory modeling was used to identify the patterns of opioid use (any or strong) among participants age 65+. We used multivariable logistic regression with backward selection to evaluate demographics and comorbidities as potential predictors of trajectory membership.

**Results:**

Among 13,059 participants, four trajectories were identified for the use of both any opioids and strong opioids (minimal-users, incident chronic-users, discontinuing-users, and prevalent chronic-users). For any opioids, female sex (adjusted odds ratio = 1.23; 95% confidence interval = 1.03–1.46), black vs. white (1.47; 1.18–1.82), year of education (0.96; 0.94–0.99), type of residence (independent group vs. private: 1.77; 1.38–2.26, care facility vs. private: 1.89; 1.20–2.97), hypertension (1.44; 1.20–1.72), cardiovascular disease (1.30; 1.09–1.55), urinary incontinence (1.45; 1.19–1.78), dementia (0.73; 0.57–0.92), number of medications (1 to 4 vs. none: 0.48; 0.36–0.64, 5 or more vs. none: 0.67; 0.50–0.88), and antidepressant agent (1.38; 1.14–1.67) were associated with incident chronic-use vs. non-use. For strong opioids, female sex (1.27; 1.04–1.56), type of residence (independent group vs. private: 1.90; 1.43–2.53, care facility vs. private: 2.37; 1.44–3.90), current smoking (1.68; 1.09–2.60), hypertension (1.49; 1.21–1.83), urinary incontinence (1.45; 1.14–1.84), dementia (0.73; 0.55–0.97), number of medications (1 to 4 vs. none: 0.46; 0.32–0.65, 5 or more vs. none: 0.59; 0.42–0.83), and antidepressant agent (1.55; 1.24–1.93) were associated with incident chronic-use vs. non-use.

**Conclusion:**

Given that chronic opioid use was more prevalent in participants who were more vulnerable (i.e., older age, with multiple comorbidities, and polypharmacy), further studies should evaluate the safety and efficacy of using opioids in this population.

## Introduction

Over 50% of the elderly population reported pain in the United States (US) in 2011, and about 75% of those reported pain in multiple sites [1]. Although chronic pain is prevalent in older adults, appropriate treatment is challenging for this population due to the high rate of polypharmacy and potential of adverse events [2]. Older adults with dementia may be especially vulnerable due to inherent difficulties in assessing and treating pain [3–5]. Long-term (≥90 days) opioid prescriptions have dramatically increased over the past decade, though the effectiveness of this therapy for chronic pain is yet to be established [6, 7]. The prevalence of long-term opioid use in US adults increased from 1.8% in 1999–2000 to 5.4% in 2013–2014 [8]. Among these long-term opioid users, 25% were adults age 65 years or older [8]. Opioid-related negative outcomes, such as addiction, misuse, and overdose deaths, have also risen [9–12]. Long-term opioid use has also been associated with opioid overdose-related hospitalization in older adults [13].

A recent study in Australia showed that opioid initiation with a transdermal formulation, higher oral morphine equivalents, older age, history of mental health comorbidities, use of non-opioid analgesics, and use of benzodiazepines were the predictors of persistent prescription opioid in adults 18 years and older [14]. A prospective study with participants in a large nonprofit health care system in Washington State reported that patients’ expectations of long-term opioid use was the main predictor of using opioids 30 or more days [15]. Although several studies reported the predictors of chronic opioid use in different populations, is the evidence is still limited regarding predictors of long-term opioid use in older adults in the US population. Older adults are more sensitive to negative outcomes (e.g., cognitive impairment, falls) from opioids, in part due to age-related decreases in liver and kidney function and polypharmacy [2, 9, 10, 12]. The Centers for Disease Control and Prevention (CDC) recently issued guidelines aimed at improving the safety and effectiveness of chronic pain treatment [16, 17]. These guidelines recommend increasing monitoring to minimize the risks of opioids in older adults, yet lack detailed guidance on opioid prescribing [16, 17]. Identifying the characteristics associated with opioid use in older adults can help identify factors that could improve risk-benefit assessment and prevent inappropriate use. Therefore, the purpose of this study was to investigate patterns of longitudinal opioid utilization in older adults using group-based trajectory models and to identify predictors associated with the trajectories indicating chronic use.

## Methods

### Study participants

**Fig 1:**
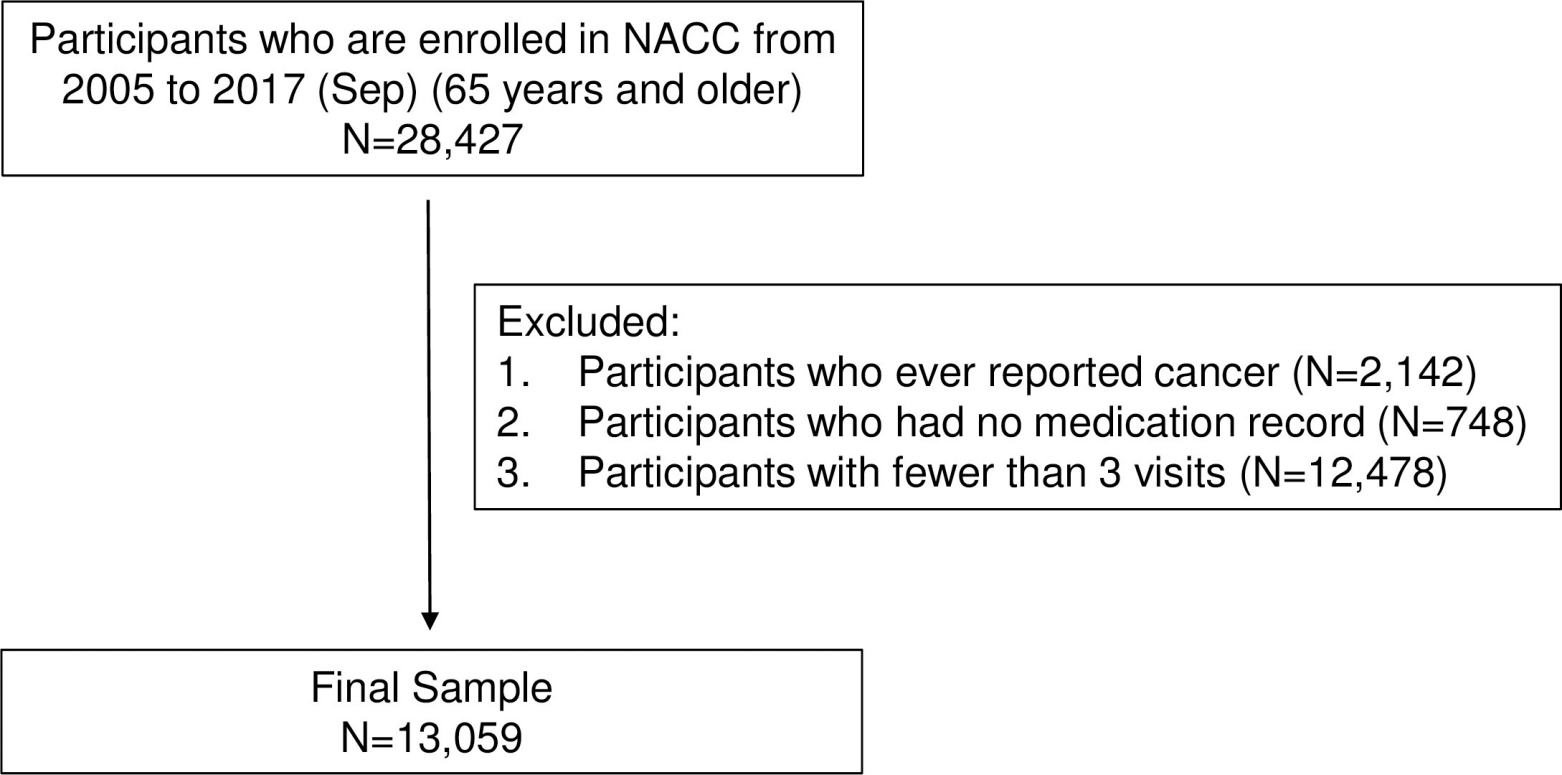
Sample selection flowchart.

Study data were drawn from the National Alzheimer’s Coordinating Center’s (NACC) Uniform Data Set (UDS), which comprises participants enrolled in longitudinal studies at National Institute on Aging-funded Alzheimer’s Disease Centers (ADC) throughout the US. Participants included subjects with cognitive status ranging from normal to dementia that are recruited through clinician referral, self-referral by patients or family members, active recruitment, and volunteers. Data from subjects and their study partners (co-participants) are collected annually by trained clinicians and other ADC research personnel until they are deceased or decline to participate. Data collected at initial and annual follow-up visits include sociodemographic characteristics, family history, medical history, neurological evaluations, and medication use information [18–20]. For this study, we included participants from 38 ADCs with data available in the September 2017 UDS data freeze, meeting the following inclusion criteria: (1) 65 years or older at their initial UDS visit, and (2) medication data recorded at every visit. Participants with fewer than three visits were excluded to facilitate assessing trajectory trends with quadratic components; in addition participants with cancer history [21], were also excluded given that opioid medications are highly prevalent in this population (Fig 1). ADC study procedures are approved by local Institutional Review Boards (IRBs), and all participants provided written informed consent. Research using the NACC database is approved by the University of Washington IRB. Because the NACC data are de-identified, no additional IRB approval was necessary for this secondary data analysis.

### Opioid use assessment

Medication information was provided by the participant and/or the caregiver/legally authorized representative and was based on each participant’s reported medication use within two weeks of each study visit. In assessing opioid use, opioid medications used as antitussives were not considered. Participants were considered to be “any opioid” users if they reported use of any opioid analgesic medications, and “strong opioid” users were defined among any opioid users if they reported use of opioid analgesics stronger than or equal to morphine’s potency [22, 23] (e.g., buprenorphine, fentanyl, hydrocodone, hydromorphone, methadone, morphine, opium, oxycodone, oxymorphone) (S1 Table).

### Participant characteristics

Baseline characteristics of interest were recorded at the participant’s initial UDS visit. Demographic information included age at enrollment (reference category [ref]: 65–74 years), sex (ref: male), race (ref: white), years of education, and type of residence (ref: single- or multiple-family private dwelling). Self-reported medical history information included current smoking, as well as ever-history of alcohol abuse, and other abused substances; hypertension, diabetes, urinary incontinence, and cardiovascular conditions. Medication information included number of medications reported (excluding opioid analgesics); use of nonsteroidal anti-inflammatory medication (NSAID), antidepressant agent, antipsychotic agent (including miscellaneous antipsychotics, psychotherapeutic combinations, phenothiazine psychotics, thioxanthenes, and atypical antipsychotics), and anxiolytic, sedative, or hypnotic agent (including barbiturates and benzodiazepines, and miscellaneous anxiolytics, sedatives, and hypnotics). Reference category for all medical history and medication variables was the absence of condition or medication use. Clinician-determined agitation (ref: no agitation) and cognitive status (ref: no dementia) were also included in the analysis [see S2 Table for detailed descriptions].

### Statistical analysis

Group-based trajectory models (GBTM) [24, 25] were used to identify participants with similar longitudinal patterns of opioid analgesic use. With this approach, latent trajectories are estimated by the model, and every individual is assigned a probability of belonging to each trajectory, with total probability of membership summing up to 1.0; we used maximum probability assignment to determine group membership. The shapes of each trajectory are defined by polynomial terms (cubic, quadratic, or linear). Since the time scale was study time, and participants could have up to 12 visits, follow-up was truncated when more than 95% of participants did not have data available for a particular visit. As a result, data from visits 11 and 12 were not included in the analysis.

Models considering between 2 and 6 trajectories were fit to the data, and the optimal final model was determined by the Bayesian Information Criterion (BIC) with the least negative value [26, 27]. In addition, for judging model adequacy, we used the approach proposed by Nagin that the average posterior probability of membership in the assigned group is greater than 0.7 [26, 27].

Once optimal GBTM models were selected, we assessed face validity by tabulating the proportion of total study visits in each trajectory group where opioid analgesic use was reported (e.g., participants assigned to the higher use groups should have higher proportion of visits where opioid analgesic use was reported). We then used multivariable logistic regression with backward selection to identify participant characteristics significantly associated with trajectory group membership. Our preliminary analyses indicated that there were participants who could be described as chronic users, such that they reported using the drugs at most visits. Since our primary interest was in identifying risk factors for this group of participants, the outcome for the logistic models was membership in a chronic user group vs. membership in a group that did not use opioid analgesics chronically.

Participants with missing values, including “unknown”, were excluded from this analysis with the exception of type of residence, which had a large number of participants in the “unknown or other” category (>600). Here, we treated unknown/other as a category. Adjusted odds ratios (OR_adj_) with 95% confidence intervals (CI) were obtained from the full and reduced models. To identify the best fitting logistic models, Akaike’s Information Criterion (AIC) values were compared between full and reduced models. All data analyses were conducted using SAS 9.4, and 0.05 was set as the significance level. PROC TRAJ [24] was used to estimate GBTM, and PROC LOGISTIC was used to fit the logistic regression models.

## Results

A total of 13,059 participants were included in our analyses after applying inclusion and exclusion criteria (Fig 1). The mean (SD) number of follow-up visits was 5.4 (2.2), ranging between 3 and 10 visits. The mean (SD) baseline age was 75.8 (6.9) years. The majority of participants were female (56.7%), white (83.1%), and resided in private dwellings (89.9%). The most common comorbidity was hypertension (55.0%), and 55.8% of participants reported taking 5 or more medications. At the initial visit, there were 498 (3.8%) users of any opioid and 284 (2.2%) users of strong opioids (Tables 1 and 2).

**Table 1:** Baseline characteristics across trajectory groups of any opioid use.

Baseline Characteristics	Pattern of Any Opioid Use			
	Minimal* (N = 11,806)	Discontinuing (N = 287)	Incident chronic (N = 657)	Prevalent chronic (N = 309)
Baseline age				
65–74	5,479 (46.4)	131 (45.6)	266 (40.5)	132 (42.7)
75–84	4,976 (42.2)	108 (37.6)	294 (44.8)	121 (39.2)
85+	1,351 (11.4)	48 (16.7)	97 (14.8)	56 (18.1)
Female	6553 (55.5)	203 (70.7)	423 (64.4)	226 (73.1)
Race				
White	9,869 (83.7)	216 (75.5)	508 (77.6)	236 (76.6)
Black	1,453 (12.3)	59 (20.6)	131 (20.0)	67 (21.8)
Other^a^	464 (3.9)	11 (3.9)	16 (2.4)	5 (1.6)
Education, mean (SD)	15.3 (3.4)	14.5 (3.6)	14.6 (3.4)	14.5 (3.4)
Type of Residence				
Private^b^	10,371 (87.9)	247 (86.1)	531 (80.8)	243 (78.6)
Independent group^c^	854 (7.2)	28 (9.8)	88 (13.4)	44 (14.2)
Care facility^d^	228 (1.9)	5 (1.7)	25 (3.8)	14 (4.5)
Unknown	353 (3.0)	7 (2.4)	13 (2.0)	8 (2.6)
Current smoking	377 (3.2)	18 (6.3)	26 (4.0)	17 (5.5)
Ever alcohol abuse	513 (4.4)	19 (6.6)	31 (4.7)	19 (6.2)
Ever other abused substances	63 (0.5)	4 (1.4)	4 (0.6)	5 (1.6)
Agitation	652 (5.5)	10 (3.5)	31 (4.7)	16 (5.2)
Ever hypertension	6,326 (53.7)	200 (69.7)	432 (65.9)	205 (66.6)
Ever diabetes	1,401 (11.9)	54 (18.8)	104 (15.8)	59 (19.2)
Ever cardiovascular disease	2,953 (25.3)	82 (28.9)	214 (32.8)	103 (33.6)
Ever urinary incontinence	1,742 (14.8)	79 (27.5)	147 (22.4)	62 (20.1)
Dementia diagnosis	2,047 (17.3)	41 (14.3)	103 (15.7)	38 (12.3)
Number of medications^e^				
0	898 (7.6)	6 (2.1)	76 (11.6)	10 (3.2)
1 to 4	4,489 (38.0)	58 (20.2)	170 (25.9)	61 (19.7)
5 or more	6,419 (54.4)	223 (77.7)	411 (62.6)	238 (77.0)
Antidepressant agent	2,645 (22.4)	109 (38.0)	184 (28.0)	121 (39.2)
Antipsychotic agent	292 (2.5)	4 (1.4)	24 (3.7)	11 (3.6)
Anxiolytic, sedative, or hypnotic agent	1,134 (9.6)	64 (22.3)	83 (12.6)	73 (23.6)
NSAID use	4,022 (34.1)	116 (40.4)	219 (33.3)	143 (46.3)
Any opioid use	121 (1.0)	162 (56.5)	9 (1.4)	206 (66.7)
Strong opioid use	67 (0.6)	90 (31.4)	6 (0.9)	121 (39.2)

(All results presented are N (%) unless otherwise noted). Abbreviations: SD, standard deviation; NSAID, nonsteroidal anti-inflammatory medication. Note

a = American Indian, Alaska Native, Native Hawaiian, Other Pacific Islander, Asian, or Other

b = single-or multiple family private living

c = retirement community, or independent group living

d = assisted living, nursing home, or hospital

e = number of opioids was excluded from the total number of medications; the minimal-use group includes participants who reported no use or low use over time.

**Table 2:** Baseline characteristics across trajectory groups of strong opioid use.

Baseline Characteristics	Pattern of strong opioid use			
	Minimal (N = 12,317)	Discontinuing (N = 116)	Incident chronic (N = 444)	Prevalent chronic (N = 182)
Baseline age				
65–74	5,699 (46.3)	57 (49.1)	176 (39.6)	76 (41.8)
75–84	5,192 (42.2)	40 (34.5)	197 (44.4)	70 (38.5)
85+	1,426 (11.6)	19 (16.4)	71 (16.0)	36 (19.8)
Female	6,904 (56.1)	79 (68.1)	289 (65.1)	133 (73.1)
Race				
White	10,232 (83.2)	92 (80.0)	362 (81.7)	143 (78.6)
Black	1,584 (12.9)	20 (17.4)	70 (15.8)	36 (19.8)
Other^a^	479 (3.9)	3 (2.6)	11 (2.5)	3 (1.7)
Education, mean (SD)	15.2 (3.4)	14.6 (3.7)	14.9 (3.4)	14.6 (3.1)
Type of Residence				
Private^b^	10,806 (87.7)	99 (85.3)	351 (79.1)	136 (74.7)
Independent group^c^	911 (7.4)	12 (10.3)	64 (14.4)	27 (14.8)
Care facility^d^	236 (1.9)	4 (3.5)	20 (4.5)	12 (6.6)
Unknown	364 (3.0)	1 (0.9)	9 (2.0)	7 (3.9)
Current smoking	391(3.2)	10 (8.7)	23 (5.2)	14 (7.7)
Ever alcohol abuse	537 (4.4)	13 (11.2)	20 (4.5)	12 (6.6)
Ever other abused substances	65 (0.5)	2 (1.7)	6 (1.4)	3 (1.7)
Agitation	672 (5.5)	4 (3.5)	26 (5.9)	7 (3.9)
Ever hypertension	6,673 (54.3)	84 (72.4)	287 (64.8)	119 (65.4)
Ever diabetes	1,505 (12.3)	23 (19.8)	62 (14.0)	28 (15.4)
Ever cardiovascular disease	3,112 (25.5)	40 (34.8)	139 (31.5)	61 (33.9)
Ever urinary incontinence	1,857 (15.1)	37 (31.9)	101 (22.8)	35 (19.2)
Dementia diagnosis	2,124 (17.2)	16 (13.8)	69 (15.5)	20 (11.0)
Number of medications^e^				
0	936 (7.6)	1 (0.9)	50 (11.3)	3 (1.7)
1 to 4	4,599 (37.3)	21 (18.1)	119 (26.8)	39 (21.4)
5 or more	6,782 (55.1)	94 (81.0)	275 (61.9)	140 (76.9)
Antidepressant agent	2,795 (22.7)	51 (44.0)	138 (31.1)	75 (41.2)
Antipsychotic agent	307 (2.5)	3 (2.6)	16 (3.6)	5 (2.8)
Anxiolytic, sedative, or hypnotic agent	1,219 (9.9)	29 (25.0)	56 (12.6)	50 (27.5)
NSAID	4,235 (34.4)	42 (36.2)	141 (31.8)	82 (45.1)
Any opioid use	259 (2.1)	81 (69.8)	28 (6.3)	130 (71.4)
Strong opioid use	82 (0.7)	78 (67.2)	3 (0.7)	121 (66.5)

(All results presented are N (%) unless otherwise noted). Abbreviations: SD, standard deviation; NSAID, nonsteroidal anti-inflammatory medication. Note

a = American Indian, Alaska Native, Native Hawaiian, Other Pacific Islander, Asian, or Other

b = single-or multiple family private living

c = retirement community, or independent group living

d = assisted living, nursing home, or hospital

e = number of opioids was excluded from the total number of medications.

**Fig 2:**
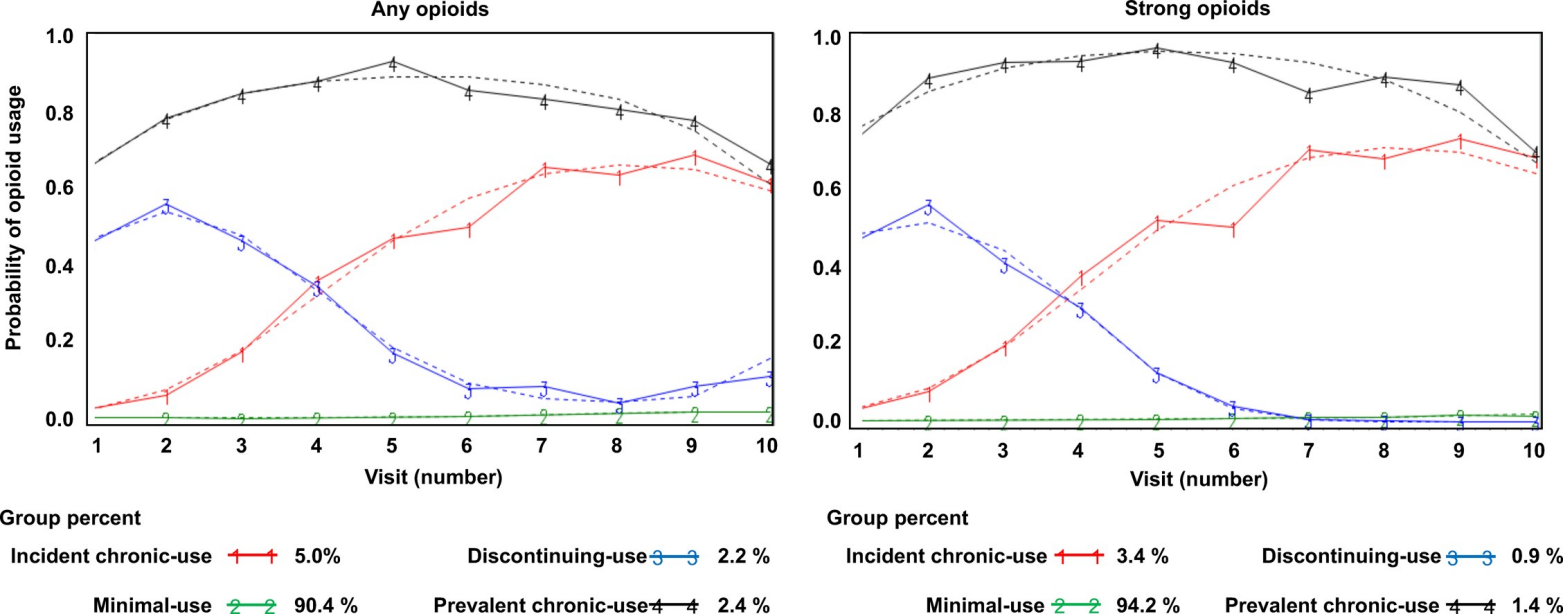
Estimated group-based trajectories for any opioid and strong opioid use in National Alzheimer’s Coordinating Center (NACC) participants (2005–2017).

Using GBTM, four trajectories were identified for both any opioid use and strong opioid use (Fig 2). The optimal number of trajectories was determined based on the BIC in combination with the requirement that the average posterior probability in all assigned trajectory groups was at least 0.70 [26, 27]. The shapes of the trajectories for any opioid use were quadratic or cubic, and the parameter estimates of the quadratic or cubic function for each trajectory were all statistically significant (S3 Table). For strong opioid use, the shapes of the trajectories were all quadratic, and the parameter estimates of the quadratic function for each trajectory were significant in 3 of the 4 groups. The final optimal models were adequate based on the criterion of the average posterior probability [26, 27] (S3 Table).

### GBTM for any opioids

A majority of participants (90.4%) were assigned to the “minimal-users” group, meaning they reported no use or low use over time. Participants (5.0%) who did not report opioid use at their first visit, but initiated use during the study period and continued their use during follow-up were assigned to the “incident chronic-users” group. “Discontinuing-users” were participants who used opioids at the first visit but discontinued during follow-up (2.2%). “Prevalent chronic-users” (2.4%) were participants who reported opioid use at baseline and consistently during follow-up (Fig 2). The median percentage (IQR) of UDS visits with any opioid use were 0% (0–0), 33.3% (25.0–40.0), 40.0% (33.3–60.0), and 85.7% (75.0–100) for minimal-users, incident chronic-users, discontinuing-users, and prevalent chronic-users, respectively. Participant characteristics for each group are presented in Table 1.

### GBTM for strong opioids

Similar trajectories—minimal-users (94.3%), incident chronic-users (3.4%), discontinuing-users (0.9%), and prevalent chronic-users (1.4%)—were identified for use of strong opioids. The median percentage (IQR) of UDS visits with strong opioid use were 0% (0–0), 33.3% (25.0–40.0), 50.0% (33.3–60.0), and 83.3% (70.0–100.0) for minimal-users, incident chronic-users, discontinuing-users, and prevalent chronic-users, respectively. Participant characteristics for each group are presented in Table 2.

### Predictors of prevalent chronic-use trajectory membership

Multivariable logistic regression models were used to identify t predictors of prevalent chronic-use trajectory membership compared to discontinuing-users or minimal-users for both, any and strong opioids. All variables listed in Table 1 were considered for inclusion (full model) (S4 and S5 Tables), and the reduced models are reported in Tables 3 and 4. After backward selection, urinary incontinence was negatively associated with prevalent chronic-use vs. discontinuing-use for both any opioids (OR_adj_ = 0.66 [95% CI = 0.45, 0.98]) and strong opioids (0.45 [0.25, 0.80]).

**Table 3:** Predictors associated with chronic-use (prevalent or incident) vs. discontinuing-use or minimal-use of any opioid in multivariable logistic regression model (reduced models adjusted for the covariates retained by backward selection).

	Prevalent chronic-use		Incident chronic-use	
	vs. discontinuing-use^a^	vs. minimal use^b^	vs. discontinuing-use^c^	vs. minimal-use^d^
Baseline age				
65–74	-	Ref.	-	-
75–84	-	1.11 (0.85, 1.44)	-	-
85+	-	1.83 (1.28, 2.61)	-	-
Female vs. male	-	1.76 (1.35, 2.30)	0.72 (0.52, 0.98)	1.23 (1.03, 1.46)
Race				
White	-	Ref.	-	Ref.
Black	-	1.92 (1.41, 2.61)	-	1.47 (1.18, 1.82)
Other	-	0.44 (0.18, 1.08)	-	0.62 (0.37, 1.04)
Education (1-year difference)	-	0.95 (0.91, 0.98)	-	0.96 (0.94, 0.99)
Type of Residence				
Private	-	Ref.	-	Ref.
Independent group	-	1.74 (1.21, 2.49)	-	1.77 (1.38, 2.26)
Care facility	-	2.02 (1.07, 3.83)	-	1.89 (1.20, 2.97)
Unknown	-	0.88 (0.42, 1.81)	-	0.66 (0.37, 1.19)
Hypertension	-	-	-	1.44 (1.20, 1.72)
Diabetes	-	1.44 (1.05, 1.97)	-	-
Cardiovascular disease	-	-	-	1.30 (1.09, 1.55)
Urinary incontinence	0.66 (0.45, 0.98)	-	-	1.45 (1.19, 1.78)
Number of medications				
None	-	Ref.	Ref.	Ref.
1 to 4	-	1.33 (0.65, 2.71)	0.26 (0.11, 0.62)	0.48 (0.36, 0.64)
5 or more	-	2.52 (1.25, 5.08)	0.17 (0.07, 0.39)	0.67 (0.50, 0.88)
Antidepressant agent	-	1.89 (1.46, 2.44)	-	1.38 (1.14, 1.67)
Anxiolytic, sedative, or hypnotic agent	-	2.26 (1.69, 3.02)	0.57 (0.39, 0.83)	-
NSAID	-	1.36 (1.06, 1.75)	-	-
Dementia	-	0.46 (0.32, 0.68)	-	0.73 (0.57, 0.92)

Note: Where the reference category is not specified, the comparison is either yes vs. no or ever vs. never.

a = Number of observations used in the model is 578 (prevalent chronic-user: 298 and discontinuing-user: 280)

b = Number of observations used in the model is 11,458 (prevalent chronic-user: 298 and minimal-users: 11,458)

c = Number of observations used in the model is 920 (incident chronic-user: 640 and discontinuing-user: 280)

d = Number of observations used in the model is 12,098 (incident chronic-user: 640 and minimal-users: 11,458

**Table 4:** Predictors associated with chronic-use (prevalent or incident) vs. discontinuing-use or non-use of strong opioids in multivariable logistic regression model (reduced models adjusted for the covariates retained by backward selection).

	Prevalent chronic-use		Incident chronic-use	
	vs. discontinuing-use^a^	vs. non-users^b^	vs. discontinuing-use^c^	vs. non-users^d^
Baseline age				
65–74	-	Ref.	-	-
75–84	-	1.13 (0.80, 1.59)	-	-
85+	-	2.10 (1.34, 3.28)	-	-
Female vs. male	-	1.71 (1.22, 2.40)	-	1.27 (1.04, 1.56)
Race				
White	-	Ref.	-	-
Black	-	1.97 (1.34, 2.91)	-	-
Other	-	0.54 (0.17, 1.73)	-	-
Type of Residence				
Private	Ref.	Ref.	-	Ref.
Independent group	2.20 (1.00, 4.85)	1.68 (1.06, 2.65)	-	1.90 (1.43, 2.53)
Care facility	3.14 (0.83, 11.91)	3.46 (1.73, 6.94)	-	2.37 (1.44, 3.90)
Unknown	6.53 (0.77, 55.73)	1.32 (0.60, 2.87)	-	0.67 (0.33, 1.36)
Current smoking	-	2.34 (1.31, 4.16)	-	1.68 (1.09, 2.60)
Alcohol abuse	-	-	0.44 (0.20, 1.00)	-
Hypertension	-	-	-	1.49 (1.21, 1.83)
Urinary incontinence	0.45 (0.25, 0.80)	-	-	1.45 (1.14, 1.84)
Dementia	-	0.39 (0.23, 0.65)	-	0.73 (0.55, 0.97)
Number of medications				
None	-	Ref.	Ref.	Ref.
1 to 4	-	2.59 (0.79, 8.45)	0.12 (0.02, 0.95)	0.46 (0.32, 0.65)
5 or more	-	4.89 (1.53, 15.65)	0.07 (0.01, 0.52)	0.59 (0.42, 0.83)
Antidepressant agent	-	1.89 (1.36, 2.63)	-	1.55 (1.24, 1.93)
Anxiolytic, sedative, or hypnotic agent	-	2.51 (1.76, 3.57)	0.53 (0.31, 0.90)	-
NSAID	1.68 (1.01, 2.78)	-	-	-

Note: Where the reference category is not specified, the comparison is either yes vs. no or ever vs. never.

a = Number of observations used in the model is 290 (prevalent chronic-user: 178 and discontinuing-user: 112)

b = Number of observations used in the model is 12,131 (prevalent chronic-user: 178 and minimal-users: 11,953)

c = Number of observations used in the model is 545 (incident chronic-user: 433 and discontinuing-user: 112)

d = Number of observations used in the model is 12,386 (incident chronic-user: 433 and minimal-users: 11,953)

Several factors emerged as significant predictors of prevalent chronic-use vs. minimal-use in both models (any opioid and strong opioids): age (any opioid: 1.83 [1.28, 2.61]; strong opioids:2.10 [1.34, 3.28]), female sex (1.76 [1.35, 2.30]; 1.71[1.22, 2.40]), black vs. white (1.92 [1.41, 2.61]; 1.97 [1.34, 2.91]), independent group living vs. private living (1.74 [1.21, 2.49]; 1.68 [1.06, 2.65]), care facility living vs. private living (2.02 [1.07, 3.83]; 3.46 [1.73, 6.94]), 5 or more medications vs. none (2.52 [1.25, 5.08]; 4.89 [1.53, 15.65]), use of antidepressant agent (1.89 [1.46, 2.44]; 1.89 [1.36, 2.63]), use of anxiolytic, sedative, or hypnotic agent (2.26 [1.69, 3.02]; 2.51 [1.76, 3.57]), and dementia (0.46 [0.32, 0.68]; 0.39 [0.23, 0.65]) (Tables 3 and 4).

### Predictors of incident chronic-use trajectory memberships

Four multivariable logistic regression models were used to identify predictors associated with incident chronic-users compared to discontinuing-users or minimal-users for both any and strong opioid groups. Use of anxiolytic, sedative, or hypnotic agent (any opioids: 0.57 [0.39, 0.83]; strong opioids: 0.53 [0.31, 0.90]), 1 to 4 medications vs. none (0.26 [0.11, 0.62]; 0.12 [0.02, 0.95]), and 5 or more medications vs. none (0.17 [0.07, 0.39]; 0.07 [0.01, 0.52]) were significant predictors of incident chronic-use vs. discontinuing-use in both models (Tables 3 and 4).

Several factors emerged as significant predictors of incident chronic-use vs. minimal-use in both models (any opioid and strong opioids): female sex (any opioid: 1.23 [1.03, 1.46]; strong opioids: 1.27 [1.04, 1.56]), independent group living vs. private living (1.77 [1.38, 2.26]; 1.90 [1.43, 2.53]), care facility living vs. private living (1.89 [1.20, 2.97]; 2.37 [1.44, 3.90])hypertension (1.44 [1.20, 1.72]; 1.49 [1.21, 1.83]), urinary incontinence (1.45 [1.19, 1.78]; 1.45 [1.14, 1.84]), use of antidepressant agent (1.38 [1.14, 1.67]; 1.55 [1.24, 1.93]), 1 to 4 medications vs. none (0.48 [0.36, 0.64]; 0.46 [0.32, 0.65]), 5 or more medications vs. none (0.67 [0.50, 0.88]; 0.59 [0.42, 0.83]) and dementia (0.73 [0.57, 0.92]; 0.73 [0.55, 0.97]) (Tables 3 and 4).

## Discussion

This study investigated the patterns of opioid analgesics (any opioid or strong opioids) use and identified predictors of inclusion in different use trajectories over 10 years of follow-up in older adults. The prevalence of any opioid use (3.8%) at enrollment was lower than that reported in a previous study (6.5%) using National Health and Nutritional Examination Survey (NHANES) from 1999 to 2014 [8]. In addition, the prevalence of any opioid use in our study was lower than that reported in other countries. A previous study from Canada has reported that the prevalence of prescription opioid use was 16.7% in the population aged 65+ in 2009 [28]. A recent study of Australians conducted by Lalic *et al*. has reported that the prevalence of prescription opioid analgesic use in people without cancer (ages 18–99 years) was 15.37% in 2016–2017 [29]. Another study has examined that 14.1% of residents (aged 65+) in the State of Victoria, Australia, filled the prescription of oxycodone in 2013 [30]. This could be due to the different definition of identifying opioid use (reported medications used within two weeks of visit vs. prescription opioid use in the past 30 days) and using different study population. Our study identified four longitudinal trends—minimal-users, incident chronic-users, discontinuing-users, and prevalent chronic-users—for use of both any and strong opioids. We found that participants who were older, female, black, residing in independent group living or care facilities, or taking antidepressant agents were more likely to be chronic-users compared to minimal-users in both the “any opioid” and “strong opioid” user groups. These results are consistent with previous studies that reported that older adults and women experience pain more frequently than younger adults and men [13, 31–33], and that older women have a higher prevalence of long-term opioid use [34]. Also, previous studies have shown that long-term opioid use is highly prevalent in nursing home residents compared to people in a community setting [35], and having depression was associated with long-term opioid use in older adults [13].

We found that taking anxiolytic, sedative, or hypnotic agents (including barbiturates and benzodiazepines) was significantly associated with prevalent chronic-use in both the any opioid and strong opioid user groups compared to minimal-use. We also observed that the prevalence of taking benzodiazepines was higher in prevalent chronic-users (2.9%) than in minimal-users (0.7%). In a recent study including adult participants of the NHANES, long-term use of opioids was associated with concurrent benzodiazepine use [8]. Similarly, a study from Australia reported that previous use of benzodiazepines was one of the predictors of persistent opioid use [14]. Considering the overdose risk of co-prescribing benzodiazepine and opioids [36], the CDC guidelines suggest avoiding the use of opioids and benzodiazepines together [16, 17]. Therefore, further studies are needed to investigate the effect of using opioids and benzodiazepines together on opioid-related adverse outcomes in older adults.

We found that patients with dementia were less likely to become chronic users of either any or strong opioids compared to non-users. This trend might be due to inherent difficulties in assessing and treating pain in these patients [4, 5], as well as potential concerns about the added burden of cognitive impairment and risk of other adverse events from opioids. Given the concern about serious problems (e.g., depression, anxiety, and agitation) that could result from under-treating pain in older adults [33, 37–40], future studies are required to thoroughly address the patterns of opioid use in patients with dementia.

Reporting a higher number of medications was positively associated with prevalent chronic-use of both any opioid and strong opioids; however, with respect to incident chronic-use, the results showed that participants with higher number of medications were less likely to be incident chronic-users compared to discontinuing-users or minimal-users. Since ADC participants may be more likely to receive medical care than the general population through their contacts with ADC clinicians, there is a possibility that the participants with polypharmacy were monitored more closely with regard to newly prescribed opioids. Thus, this result may not be generalizable to all older adults in the US.

Neither comorbidities nor number of medications significantly predicted prevalent chronic-use vs. discontinuing use. A recent prospective study concluded that neither baseline chronic pain risk score nor depression were predictors of long-term opioid use; rather, a patient’s expectation of long-term opioid use was the strongest predictor [15]. In a recent study, long-term opioid use was significantly associated with physicians who have high-intensity of prescribing opioids [41]. We also examined group percentages of discontinuing- and chronic-users among the different ADCs (S6 Table) and observed that some centers had a higher proportion of discontinuing users than others. Thus, it is possible that clinicians at different ADCs implement varying approaches in the management of pain and the de-escalation and discontinuation of opioids in participants who use these medications chronically. Future studies that include other factors (e.g., clinician characteristics or patient’s expectation) are needed to fully understand how the prevalent chronic opioid-user group is different from the discontinuing group.

This study has several limitations. First, because opioids were identified by reported medications used within two weeks of UDS visit, we could not classify participants by continuous long-term use of opioids. Given the short exposure window, participants could be misclassified if they used opioids only between visits. However, with up to 10 years of annual assessments, we believe that we have meaningful information regarding longitudinal use patterns. Additionally, ADC participants tend to be highly educated, which may limit generalizability [18]. Also, participants who were excluded from the study had a higher rate of comorbidities and a higher rate of using any opioid/strong opioids at baseline (S7 Table). The selection bias from this exclusion criterion may result in an underestimate of opioid usage in this cohort. However, since the mean number of visits for each trajectory group in any opioid users was similar (minimal user: 5.38; incident chronic user: 5.75; discontinuing-user: 5.13; prevalent chronic user: 5.06), participants with less of follow up didn’t affected the participants being in their trajectory group. Finally, we did not consider time-varying covariates, which may have resulted in different associations.

In conclusion, the present study showed that potentially inappropriate opioid use was disproportionately prevalent among vulnerable NACC participants (i.e., older age, with multiple comorbidities and polypharmacy). Further studies are required to thoroughly address the risk and benefit of using opioids in older adults, and it is essential to provide evidence-based guidelines for opioid use in this population.

## Supporting information

S1 Table List of drugs included in “any opioids” and “strong opioids”. (PDF)

S2 Table Description of variables used in the study. (PDF)

S3 Table Description of estimated trajectories and number of participants in each trajectory. (PDF)

S4 Table Factors associated with chronic-use (prevalent or incident) vs. discontinuing-use and chronic-use (prevalent or incident) vs. non-use of any opioids in multivariable logistic regression model (full model). (PDF)

S5 Table Factors associated with chronic-use (prevalent or incident) vs. discontinuing-use and chronic-use (prevalent or incident) vs. non-use of strong opioids in multivariable logistic regression model (full model). (PDF)

S6 Table The frequency distribution across trajectory groups of any opioid use among Alzheimer’s Disease Centers (ADC). (PDF)

S7 Table Participant characteristics: Included participants vs. participants excluded for having fewer than 3 visits. (PDF)
