# Supplementary material for: Patterns and predictors of chronic opioid use in older adults: A retrospective cohort study
Source: PLoS One. 2019 Jan 11;14(1):e0210341. doi: 10.1371/journal.pone.0210341 (PMC6329525; doi:10.1371/journal.pone.0210341)
Supplement: S1 Table — (PDF) [file pone.0210341.s001.pdf]

S1 Table. List of drugs included in "any opioids" and "strong opioids"

Any opioids: included any opioid analgesic medications

Acetaminophen-codeine	Acetaminophen-hydrocodone'
Acetaminophen-oxycodone	Acetaminophen-tramadol'
Acetaminophen/butalbital/caffeine/ codeine	Acetaminophen/caffeine/dihydrocodeine
Asa/caffeine/propoxyphene	Aspirin/butalbital/caffeine/codeine
Aspirin/caffeine/dihydrocodeine	Aspirin/carisoprodol/codeine'
Aspirin-hydrocodone	Aspirin-oxycodone
Bupivacaine-fentanyl	Bupivacaine-hydromorphone
Buprenorphine	Codeine
Dihydrocodeine	Droperidol-fentanyl
Fentanyl	Fentanyl topical
Fentanyl-ropivacaine	Hydrocodone
Hydrocodone-ibuprofen	Hydromorphone
Meperidine	Methadone
Morphine	Opium
Oxycodone	Oxymorphone
Propoxyphene	Tramadol

Strong opioids: included opioids stronger than or equal to morphine's potency*

Acetaminophen-hydrocodone	Acetaminophen-oxycodone
Aspirin-hydrocodone	Aspirin-oxycodone
Bupivacaine-fentanyl	Bupivacaine-hydromorphone
Buprenorphine	Droperidol-fentanyl
Fentanyl	Fentanyl topical
Fentanyl-ropivacaine	Hydrocodone
Hydrocodone-ibuprofen	Hydromorphone
Methadone	Morphine
Opium	Oxycodone
Oxymorphone	

* Pasero C, McCaffery M. Pain assessment and pharmacologic management. St. Louis, MO: Mosby; 2010.
Horgas AL, Snigurska U, Farland MZ, Marsiske M. Analyzing Analgesic Medications in Community-Dwelling Older Adults. *Pain Med.* 2018
