# Supplementary material for: Patterns and predictors of chronic opioid use in older adults: A retrospective cohort study
Source: PLoS One. 2019 Jan 11;14(1):e0210341. doi: 10.1371/journal.pone.0210341 (PMC6329525; doi:10.1371/journal.pone.0210341)
Supplement: S2 Table — (PDF) [file pone.0210341.s002.pdf]

S2 Table. Description of variables used in the study

Variables	Form	Description
Baseline age	A1 subject demographics	Subject's age at visit
Female	A1 subject demographics	Subject's sex
Race	A1 subject demographics	White<br>Black or African American<br>Others (American Indian, Alaska Native, Native Hawaiian, Pacific Islander, Asian, or Other)<br>(Missing=24)
Education	A1 subject demographics	Years of education (0-36) (Missing=40)
Type of Residence	A1 subject demographics	Single- or multi- family private residence<br>Retirement community or independent group living<br>Assisted living, adult family home, boarding home, skilled nursing facility, nursing home, hospital, or hospice<br>Unknown
Smoking cigarettes	A5 subject health history	Smoked cigarettes in last 30 days (yes vs. no) (Missing=51)
Alcohol abuse	A5 subject health history	Alcohol abuse-clinically significant impairment occurring over a 12-month period manifested in one of the following area: work, driving, legal, or social (ever vs. never) (Missing=28)
Other abused substances	A5 subject health history	Other abused substances - clinically significant impairment occurring over a 12-month period manifested in one of the following area: work, driving, legal, or social (ever vs. never) (Missing=34)
Agitation	B9 Clinician Judgement of Symptoms	Subject currently manifests meaningful change in behavior – agitation (yes vs. no) (Missing=36)
Hypertension	A5 subject health history	Hypertension (ever vs. never) (Missing=35)
Diabetes	A5 subject health history	Diabetes (ever vs. never) (Missing=50)
Any cardiovascular disease	A5 subject health history	Any history of Heart attack/cardiac arrest, Atrial fibrillation, Congestive heart failure, Stroke, or Other cardiovascular disease (ever vs. never) (Missing=119)
Urinary incontinence	A5 subject health history	Incontinence – urinary (ever vs. never) (Missing=31)
Cancer	D1 clinician diagnosis<br>D2 clinician-assessed medical conditions	Presence of cancer (yes vs. no)
Number of Medications	A4 subject medications	Total number of medications reported at the participant's initial UDS visit (excluded the number of opioids use at the participant's initial visit)

Antidepressant agent	A4 subject medications	Report current use of antidepressant
Antipsychotic agent	A4 subject medications	Report current use of antipsychotic agent
Anxiolytic, sedative, or hypnotic agent	A4 subject medications	Report current use of anxiolytic, sedative, or hypnotic agent
Nonsteroidal anti-inflammatory medication	A4 subject medications	Report current use of nonsteroidal anti-inflammatory medication
Dementia	B4 global staging<br>D1 clinician diagnosis	Clinician diagnosis (NACCUDSD) of dementia plus Clinician Dementia Rating (CDR)† score ≥ 1
Use of opioids<br>(any and strong)	A4 subject medications	Medication used within two weeks of UDS visit

†CDR score 0: no impairment; 0.5: questionable impairment; 1: mild impairment; 2: moderate impairment; 3: severe impairment

Morris JC. The Clinical Dementia Rating (CDR): current version and scoring rules. *Neurology.* 1993;43(11):2412-2414.
