# Supplementary material for: Patterns and predictors of chronic opioid use in older adults: A retrospective cohort study
Source: PLoS One. 2019 Jan 11;14(1):e0210341. doi: 10.1371/journal.pone.0210341 (PMC6329525; doi:10.1371/journal.pone.0210341)
Supplement: S3 Table — (PDF) [file pone.0210341.s003.pdf]

S3 Table. Description of estimated trajectories and number of participants in each trajectory

Trajectory Group	N	Polynomial[a]	Estimates (SE)[b]	p-value[c]	Average posterior probability[d]
**Any opioid**					
Minimal-use*	11,806	Cubic	-0.0112 (0.004)	0.003	0.96
Discontinuing use	287	Cubic	0.0305 (0.006)	<0.001	0.72
Incident chronic use	657	Quadratic	-0.0847 (0.011)	<0.001	0.73
Prevalent chronic-use	309	Quadratic	-0.0807 (0.014)	<0.001	0.77
**Strong opioids**					
Minimal-use	12,317	Quadratic	0.0094 (0.015)	0.52	0.97
Discontinuing use	116	Quadratic	-0.1867 (0.074)	0.011	0.78
Incident chronic use	444	Quadratic	-0.0803 (0.012)	<0.001	0.71
Prevalent chronic-use	182	Quadratic	-0.0641 (0.017)	<0.001	0.81

Note: a=highest order of the trajectory group; b=parameter estimates of the highest order and standard error (SE); c=p-value for the highest order of the trajectory group; d=average posterior probability for the participants assigned to the trajectory group

*minimal-use refers to participants who reported no opioid use or low use over time
