# Supplementary material for: Patterns and predictors of chronic opioid use in older adults: A retrospective cohort study
Source: PLoS One. 2019 Jan 11;14(1):e0210341. doi: 10.1371/journal.pone.0210341 (PMC6329525; doi:10.1371/journal.pone.0210341)
Supplement: S4 Table — (PDF) [file pone.0210341.s004.pdf]

**S4 Table. Factors associated with chronic-use (prevalent or incident) vs. discontinuing-use and chronic-use (prevalent or incident) vs. minimal use of any opioids in multivariable logistic regression model (full model)**

| | Prevalent chronic-use | | Incident chronic-use | |
|---|---|---|---|---|
| | vs. discontinuing-use | vs. minimal-use | vs. discontinuing-use | vs. minimal-use |
| Baseline age | | | | |
| 65-74 | Ref. | Ref. | Ref. | Ref. |
| 75-84 | 1.17 (0.80, 1.72) | 1.10 (0.84, 1.43) | 1.07 (0.76, 1,50) | 1.11 (0.92, 1.33) |
| 85+ | 1.16 (0.69, 1.96) | 1.81 (1.26, 2.61) | 0.67 (0.41, 1.08) | 1.17 (0.89, 1.52) |
| Female | 1.07 (0.72, 1.59) | 1.85 (1.40, 2.43) | 0.70 (0.51, 0.98) | 1.23 (1.03, 1.46) |
| Race | | | | |
| White | Ref. | Ref. | Ref. | Ref. |
| Black | 1.23 (0.78, 1.93) | 1.80 (1.31, 2.46) | 0.82 (0.54, 1.25) | 1.47 (1.17, 1.84) |
| Other | 0.50 (0.17, 1.52) | 0.42 (0.17, 1.05) | 0.66 (0.28, 1.52) | 0.61 (0.37, 1.03) |
| Education (1-year difference) | 1.00 (0.95, 1.05) | 0.95 (0.92, 0.99) | 1.02 (0.97, 1.06) | 0.96 (0.94, 0.99) |
| Type of Residence | | | | |
| Private[a] | Ref. | Ref. | Ref. | Ref. |
| Independent group[b] | 1.73 (0.98, 3.04) | 1.69 (1.17, 2.43) | 2.00 (1.19, 3.35) | 1.69 (1.31, 2.18) |
| Care facility[c] | 2.70 (0.87, 8.33) | 1.90 (1.00, 3.64) | 2.21 (0.76, 6.42) | 1.78 (1.12, 2.84) |
| Unknown | 1.14 (0.39, 3.34) | 0.86 (0.42, 1.78) | 0.93 (0.35, 2.47) | 0.66 (0.37, 1.18) |
| Current smoking | 0.82 (0.39, 1.72) | 1.52 (0.90, 2.56) | 0.59 (0.29, 1.19) | 1.15 (0.76, 1.75) |
| Ever alcohol abuse | 0.99 (0.45, 2.17) | 1.24 (0.72, 2.12) | 0.74 (0.37, 1.46) | 1.06 (0.72, 1.58) |
| Ever other abused substances | 1.19 (0.27, 5.30) | 2.25 (0.84, 6.00) | 0.60 (0.12, 3.06) | 0.98 (0.34, 2.81) |
| Agitation | 1.59 (0.64, 3.98) | 1.04 (0.59, 1.83) | 1.08 (0.47, 2.49) | 0.77 (0.51, 1.15) |
| Ever hypertension | 0.83 (0.56, 1.23) | 1.22 (0.94, 1.59) | 0.95 (0.67, 1.34) | 1.42 (1.19, 1.71) |
| Ever diabetes | 1.03 (0.65, 1.65) | 1.38 (1.00, 1.89) | 0.83 (0.55, 1.26) | 1.10 (0.87, 1.39) |
| Ever cardiovascular disease | 1.24 (0.85, 1.80) | 1.22 (0.94, 1.58) | 1.32 (0.94, 1.84) | 1.27 (1.06, 1.52) |
| Ever urinary incontinence | 0.60 (0.40, 0.91) | 1.04 (0.77, 1.41) | 0.78 (0.55, 1.11) | 1.43 (1.17, 1.76) |
| Dementia diagnosis | 0.70 (0.40, 1.24) | 0.45 (0.30, 0.68) | 1.15 (0.73, 1.83) | 0.73 (0.57, 0.94) |
| Number of medications | | | | |
| 0 | Ref. | Ref. | Ref. | Ref. |
| 1 to 4 | 0.69 (0.22, 2.19) | 1.31 (0.64, 2.67) | 0.23 (0.09, 0.57) | 0.48 (0.36, 0.65) |
| 5 or more | 0.60 (0.19, 1.85) | 2.30 (1.13, 4.67) | 0.15 (0.06, 0.38) | 0.67 (0.50, 0.90) |
| Antidepressant agent | 1.16 (0.80, 1.68) | 1.82 (1.40, 2.37) | 0.77 (0.55, 1.08) | 1.36 (1.12, 1.66) |
| Antipsychotic agent | 2.58 (0.73, 9.11) | 1.24 (0.62, 2.50) | 2.99 (0.95, 9.42) | 1.29 (0.79, 2.10) |
| Anxiolytic, sedative, or hypnotic agent | 1.06 (0.70, 1.61) | 2.23 (1.67, 2.98) | 0.56 (0.38, 0.82) | 1.24 (0.97, 1.60) |
| NSAID | 1.47 (1.03, 2.10) | 1.37 (1.07, 1.76) | 1.03 (0.75, 1.43) | 0.93 (0.78, 1.13) |

Abbreviations: NSAID, nonsteroidal anti-inflammatory medication.

a=single-or multiple family private living; b=retirement community, or independent group living; c=assisted living, nursing home, or hospital
