# Supplementary material for: Patterns and predictors of chronic opioid use in older adults: A retrospective cohort study
Source: PLoS One. 2019 Jan 11;14(1):e0210341. doi: 10.1371/journal.pone.0210341 (PMC6329525; doi:10.1371/journal.pone.0210341)
Supplement: S5 Table — (PDF) [file pone.0210341.s005.pdf]

S5 Table. Factors associated with chronic-use (prevalent or incident) vs. discontinuing-use and chronic-use (prevalent or incident) vs. non-use of strong opioids in multivariable logistic regression model (full model)

	Prevalent chronic-use		Incident chronic-use	
	vs. discontinuing-use	vs. minimal-use	vs. discontinuing-use	vs. minimal-use
Baseline age				
65-74	Ref.	Ref.	Ref.	Ref.
75-84	1.39 (0.78, 2.51)	1.11 (0.79, 1.57)	1.20 (0.71, 2,01)	1.14 (0.92, 1.42)
85+	1.98 (0.86, 4.56)	2.04 (1.29, 3.23)	0.87 (0.42, 1.81)	1.29 (0.94, 1.77)
Female	1.12 (0.62, 2.02)	1.80 (1.26, 2.57)	0.89 (0.54, 1.47)	1.26 (1.02, 1.56)
Race				
White	Ref.	Ref.	Ref.	Ref.
Black	1.80 (0.86, 3.77)	1.73 (1.15, 2.60)	0.83 (0.42, 1.64)	1.06 (0.80, 1.42)
Other	1.18 (0.19, 7.50)	0.48 (0.15, 1.54)	1.32 (0.31, 5.65)	0.63 (0.34, 1.17)
Education (1-year difference)	1.02 (0.94, 1.11)	0.97 (0.92, 1.01)	1.05 (0.98, 1.12)	0.99 (0.96, 1.02)
Type of Residence				
Private[a]	Ref.	Ref.	Ref.	Ref.
Independent group[b]	1.88 (0.80, 4.45)	1.70 (1.07, 2.69)	2.12 (0.98, 4.57)	1.73 (1.28, 2.33)
Care facility[c]	2.43 (0.56, 10.59)	3.47 (1.71, 7.03)	1.40 (0.36, 5.43)	2.06 (1.23, 3.44)
Unknown	8.19 (0.90, 74.93)	1.32 (0.61, 2.89)	3.54 (0.40, 31.50)	0.66 (0.32, 1.35)
Current smoking	0.80 (0.31, 2.08)	2.20 (1.23, 3.95)	0.67 (0.27, 1.67)	1.67 (1.07, 2.60)
Ever alcohol abuse	0.84 (0.30, 2.40)	1.47 (0.77, 2.81)	0.51 (0.21, 1.24)	0.89 (0.54, 1.46)
Ever other abused substances	1.27 (0.16, 9.75)	1.86 (0.53, 6.57)	1.48 (0.22, 10.09)	2.38 (0.97, 5.84)
Agitation	2.01 (0.44, 9.13)	0.84 (0.37, 1.89)	2.04 (0.51, 8.14)	1.02 (0.65, 1.60)
Ever hypertension	0.62 (0.34, 1.11)	1.20 (0.85, 1.68)	0.85 (0.50, 1.44)	1.43 (1.15, 1.78)
Ever diabetes	0.83 (0.41, 1.70)	1.10 (0.71, 1.70)	0.79 (0.42, 1.48)	1.02 (0.76, 1.36)
Ever cardiovascular disease	0.95 (0.55, 1.65)	1.25 (0.90, 1.73)	1.09 (0.67, 1.78)	1.15 (0.93, 1.43)
Ever urinary incontinence	0.43 (0.23, 0.81)	0.94 (0.63, 1.40)	0.58 (0.35, 0.97)	1.39 (1.09, 1.77)
Dementia diagnosis	0.79 (0.33, 1.90)	0.39 (0.23, 0.68)	1.44 (0.70, 2.93)	0.70 (0.52, 0.95)
Number of medications				
0	Ref.	Ref.	Ref.	Ref.
1 to 4	0.63 (0.05, 7.59)	2.50 (0.76, 8.20)	0.09 (0.01, 0.76)	0.48 (0.34, 0.68)
5 or more	0.54 (0.05, 6.33)	4.00 (1.23, 13.06)	0.06 (0.01, 0.44)	0.63 (0.44, 0.91)
Antidepressant agent	1.08 (0.62, 1.89)	1.88 (1.35, 2.62)	0.76 (0.47, 1.23)	1.53 (1.22, 1.93)
Antipsychotic agent	2.42 (0.36, 16.09)	0.95 (0.36, 2.48)	2.17 (0.43, 11.02)	1.23 (0.69, 2.19)
Anxiolytic, sedative, or hypnotic agent	1.19 (0.65, 2.17)	2.53 (1.77, 3.62)	0.47 (0.27, 0.83)	1.14 (0.84, 1.54)
NSAID	1.95 (1.13, 3.36)	1.28 (0.93, 1.76)	1.21(0.74, 2.00)	0.86 (0.69, 1.08)

Abbreviations: NSAID, nonsteroidal anti-inflammatory medication.

a=single-or multiple family private living; b=retirement community, or independent group living; c=assisted living, nursing home, or hospital
