# Supplementary material for: Patterns and predictors of chronic opioid use in older adults: A retrospective cohort study
Source: PLoS One. 2019 Jan 11;14(1):e0210341. doi: 10.1371/journal.pone.0210341 (PMC6329525; doi:10.1371/journal.pone.0210341)
Supplement: S6 Table — (PDF) [file pone.0210341.s006.pdf]

S6 Table. The frequency distribution across trajectory groups of any opioid use among Alzheimer's Disease Centers (ADC)

ADC*<br>N (%)	Minimal<br>(N=11,806)	Discontinuing<br>(N=287)	Incident chronic<br>(N=657)	Prevalent<br>chronic (N=309)
289	359 (94.2)	6 (1.6)	9 (2.4)	7 (1.8)
354	357 (93.0)	7 (1.8)	14 (3.7)	6 (1.6)
490	250 (90.9)	8 (2.9)	14 (5.1)	3 (1.1)
911	17 (100.0)	0 (0)	0 (0)	0 (0)
1354	416 (89.5)	13 (2.8)	26 (5.6)	10 (2.2)
1416	393 (94.2)	8 (1.9)	9 (2.2)	7 (1.7)
2096	521 (88.0)	12 (2.0)	50 (8.5)	9 (1.5)
2289	539 (93.7)	10 (1.7)	21 (3.7)	5 (0.9)
2578	384 (88.9)	13 (3.0)	24 (5.6)	11 (2.6)
2958	232 (91.3)	4 (1.6)	15 (5.9)	3 (1.2)
3630	271 (90.6)	7 (2.3)	12 (4.0)	9 (3.0)
3697	172 (94.5)	2 (1.1)	4 (2.2)	4 (2.2)
4347	386 (96.0)	2 (0.5)	13 (3.2)	1 (0.3)
4935	395 (89.0)	15 (3.4)	24 (5.4)	10 (2.3)
4967	642 (95.3)	11 (1.6)	13 (1.9)	8 (1.2)
5310	265 (92.3)	4 (1.4)	12 (4.2)	6 (2.1)
5452	658 (93.1)	13 (1.8)	24 (3.4)	12 (1.7)
5783	561 (82.4)	27 (4.0)	54 (7.9)	39 (5.7)
5897	196 (84.9)	4(1.7)	24 (10.4)	7 (3.0)
6061	481 (96.8)	5 (1.0)	9 (1.8)	2 (0.4)
6499	170 (88.5)	4 (2.1)	12 (6.3)	6 (3.1)
6518	459 (84.8)	11 (2.0)	48 (8.9)	23 (4.3)
6713	239 (85.4)	8 (2.9)	20 (7.1)	13 (4.6)
8354	195 (84.8)	8 (3.5)	16 (7.0)	11 (4.8)
8361	487 (87.4)	21 (3.8)	32 (5.8)	17 (3.1)
8646	694 (90.0)	20 (2.5)	49 (6.2)	23 (2.9)
8658	636 (90.0)	19 (2.7)	30 (4.2)	22 (3.1)
8660	121 (91.7)	3 (2.3)	4 (3.0)	4 (3.0)
8683	291 (91.2)	7 (2.2)	12 (3.8)	9 (2.8)
8974	277 (84.7)	7 (2.1)	29 (8.9)	14 (4.3)
9637	371 (92.8)	6 (1.5)	20 (5.0)	3 (0.8)
9661	371 (94.6)	2 (0.5)	14 (3.6)	5 (1.3)

* The ADC number refers to the center number assigned by the National Alzheimer's Coordinating Center to allow for data sharing while protecting privacy and confidentiality for study participants.
