# Supplementary material for: Patterns and predictors of chronic opioid use in older adults: A retrospective cohort study
Source: PLoS One. 2019 Jan 11;14(1):e0210341. doi: 10.1371/journal.pone.0210341 (PMC6329525; doi:10.1371/journal.pone.0210341)
Supplement: S7 Table — (PDF) [file pone.0210341.s007.pdf]

S7 Table. Participant characteristics: Study participants vs. participants excluded for having fewer than 3 visits

Characteristics	Study population (N=13,059)	Excluded (N=12,478)
Baseline age		
65-74	6008 (46.01)	5465 (43.80)
75-84	5499 (42.11)	5216 (41.80)
85+	1552 (11.88)	1797 (14.40)
Female	7,405 (56.70)	7,069 (56.65)
Race		
White	10,829 (83.08)	9,788 (78.92)
Black	1,710 (13.12)	1,892 (15.25)
Other	496 (3.81)	723 (5.83)
Education, mean (SD)	15.20 (3.43)	14.57 (3.76)
Type of Residence		
Private[a]	11,392 (87.23)	10,925 (87.55)
Independent group[b]	1,014 (7.76)	667 (5.35)
Care facility[c]	272 (2.08)	658 (5.27)
Unknown	381 (2.92)	228 (1.83)
Current smoking	438 (3.37)	516 (4.16)
Ever alcohol abuse	582 (4.47)	727 (5.85)
Ever other abused substances	76 (0.58)	140 (1.13)
Agitation	709 (5.44)	1429 (11.48)
Ever hypertension	7,163 (55.00)	7,149 (57.56)
Ever diabetes	1618 (12.44)	1916 (15.40)
Ever cardiovascular disease	3,352 (25.90)	3,519 (28.59)
Urinary incontinence (ever vs. never)	2,030 (15.58)	2,692 (21.65)
Dementia diagnosis	2229 (17.07)	4236 (33.95)
Number of medications		
0	990 (7.56)	709 (5.68)
1 to 4	4,449 (36.60)	4,167 (33.39)
5 or more	7,290 (55.82)	7,602 (60.92)
Antidepressant agent	3059 (23.42)	3781 (30.30)
Antipsychotic agent	331 (2.53)	731 (5.86)
Anxiolytic, sedative, or hypnotic agent	1354 (10.37)	1604 (12.85)
Nonsteroidal anti-inflammatory medication	4500 (34.46)	4357 (34.92)
Any opioid use	498 (3.81)	624 (5.00)
Strong opioid use	284 (2.17)	398 (3.19)

(All results presented are N (%) unless otherwise noted)

a=single-or multiple family private living; b=retirement community, or independent group living; c=assisted living, nursing home, or hospital
